# A note on detecting statistical outliers in psychophysical data

**DOI:** 10.3758/s13414-019-01726-3

**Published:** 2019-05-14

**Authors:** Pete R. Jones

**Affiliations:** 1 Institute of Ophthalmology, University College London (UCL), London, EC1V 9EL UK; 2 NIHR Moorfields Biomedical Research Centre, London, EC1V 2PD UK

**Keywords:** Statistics, Cognitive neuroscience

## Abstract

This paper considers how to identify statistical outliers in psychophysical datasets where the underlying sampling distributions are unknown. Eight methods are described, and each is evaluated using Monte Carlo simulations of a typical psychophysical experiment. The best method is shown to be one based on a measure of spread known as *S*_*n*_. This is shown to be more sensitive than popular heuristics based on standard deviations from the mean, and more robust than non-parametric methods based on percentiles or interquartile range. Matlab code for computing *S*_*n*_ is included.

## The problem of outliers

**Fig. 1:**
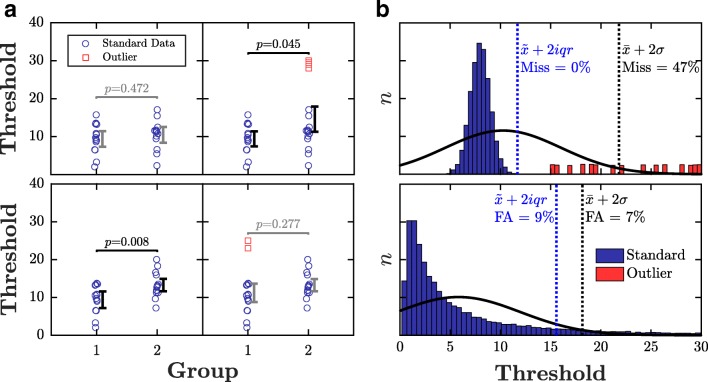
Example hypothetical data showing: **a** how the presence of statistical outliers (*red squares*) can qualitatively affect the overall pattern of results, and **b** common errors made by existing methods of outlier identification heuristics, including misses and false alarms (FA). *P* values in panel a pertain to the results of between-subject *t* tests

Statistical outliers are observations that diverge abnormally from the overall pattern of data. They are often generated by processes qualitatively distinct from the main body of data. For example, in psychophysics, spurious data can be caused by technical error, faulty transcription, or—perhaps most commonly—participants being unable or unwilling to perform the task in the manner intended (e.g., due to boredom, fatigue, poor instruction, or malingering). Whatever the cause, statistical outliers can profoundly affect the results of an experiment (Osborne & Overbay, 2004), making similar populations appear distinct (Fig. 1a, top panel), or distinct populations appear similar (Fig. 1a, bottom panel). For example, it is tempting to wonder how many ‘developmental’ differences between children and adults are due to the extreme data emanating from a small subset of badly behaved (‘non-compliant’) children.

## General approaches and outstanding questions

One way to militate against statistical outliers is to only ever use non-parametric statistics. These have a high breakdown point (Huber, 2011), and so tend to be relatively unaffected by small numbers of extreme values. In reality though, when it comes to inferential hypothesis testing, non-parametric methods are often impractical, since they are less powerful, less well understood, and less widely available than their parametric counterparts.

Alternatively, many experimenters identify and remove outliers ‘manually’, using some often unspecified process of ‘inspection’. This approach is not without merit. However, when used in isolation, manual inspection is susceptible to bias and human error, and it precludes rigorous replication or review.

Finally then, statistical outliers can be identified numerically. If the underlying sampling distribution is known a priori, then it is trivial to set a cutoff based on the likelihood of observing each data point. In most psychophysical experiments, however, the underlying sampling distribution is unknown. Indeed, it is often the very properties of this distribution that we are attempting estimate (e.g., the mean value of some variable, *x*, or its standard deviation).

When the sampling distribution is unknown, researchers are often compelled to use heuristics to identify outliers, such as “was the data point more than *N* standard deviations from the mean?” (Fig. 1b). At present, a plethora of such heuristics exist in common usage. It is unclear which method works best, and careless or unscrupulous experimenters are free to pick-and-choose whichever yields the outcome they expect or desire.

The goal of the present work was therefore to: (i) describe the methods currently available for identifying statistical outliers (in data generated from unknown sampling distributions), and (ii) use simulations to assess how well each method performs in a typical psychophysical context.

## State-of-the-art methods for identifying statistical outliers

Here we describe eight methods for identifying statistical outliers. Some of this information can also be found in a more wide ranging review by Cousineau and Chartier (2010).

### SD

*x*_*i*_=outlier if it lies more than *λ* standard deviations, *σ*, from the mean, $\bar {x}$:

1 $$ |x_{i}| > \left( \bar{x} + \lambda{}\sigma \right), $$

where *λ* typically ranges from 2 (liberal) to 3 (conservative). This is one of the most commonly used heuristics, but it has substantial flaws. Both the $\bar {x}$ and *σ* terms are easily distorted by extreme values, meaning that more distant outliers may ‘mask’ lesser ones. This can lead to false negatives (identifying outliers as genuine data; Fig. 1b, top panel). The method also assumes symmetry (i.e., attributes equal importance to positive and negative deviations from the center), whereas psychometric data are often skewed—since, for example, the process that lead to outlying data may lead to sensory abilities being disproportionately underestimated, rather than overestimated (e.g., see Section 2). The misassumption of symmetry can lead to false positives (identifying genuine data as outliers; Fig. 1b, bottom panel). Finally, while the *SD* heuristic does not explicitly require the sample distribution to be Gaussian distributed, the ± *λσ* bracket may include more or less data than expected if data are not. For example, ± 2*σ* would exclude 5% of the most extreme values when data are Gaussian, but as much as 25% otherwise (see Chebyshev’s inequality).

### GMM

*x*_*i*_=outlier if it lies more than *λ* standard deviations from the mean *of the primary component of a Gaussian Mixture Model*:

2 $$ \begin{array}{@{}rcl@{}} &&|x_{i}| > \left( \bar{x}_{1} + \lambda{}\sigma_{1} \right) \qquad where\\ &&pdf(x) = \omega {\Phi}(x; \mu_{1}, \sigma_{1}) + (1 - \omega) {\Phi}(x; \mu_{2}, \sigma_{2}). \end{array} $$

A logical extension to *SD*: The two methods are identical, except that when fitting the parameters to the data, the *GMM* model also includes a secondary Gaussian component designed to capture any outliers. This second component is not used to identify outliers per se, but instead prevents extreme values from distorting the parameter estimates of the primary component. In practice, the fit of the secondary component must be constrained to prevent it from ‘absorbing’ non-outlying points. For example, if it is suspected that some observers did not understand the task, then one might posit a second distribution with a mean constrained to a near-floor level of performance.

**Listing 1:**
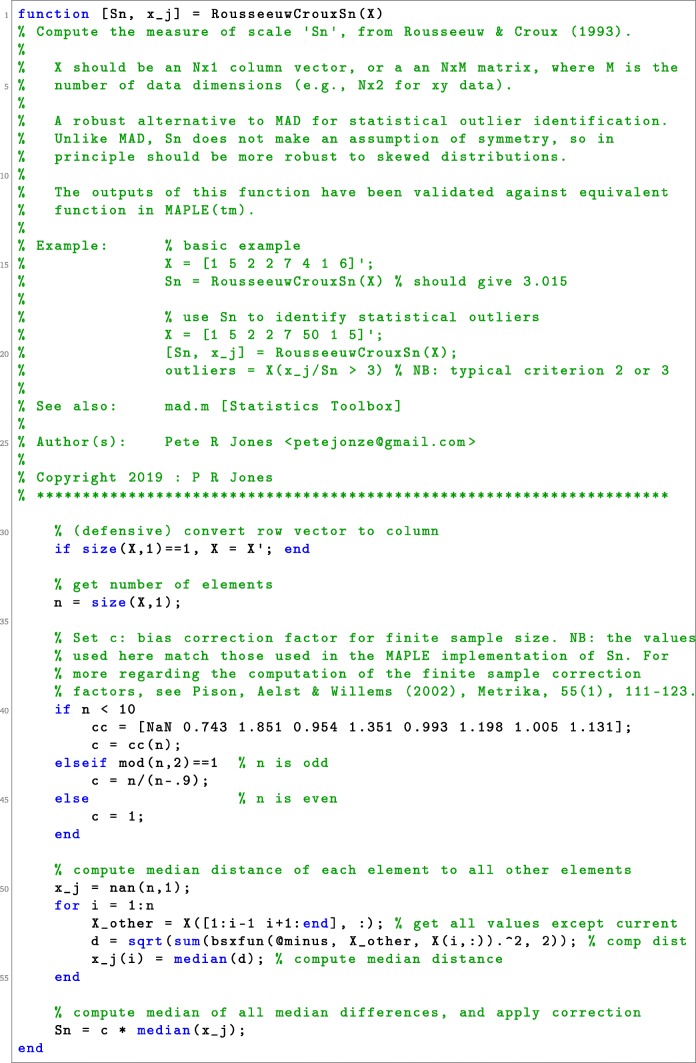
Matlab code for computing Rousseeuw & Croux’s measure of spread: *S*_*n*_

The *GMM* heuristic can be further extended by adding a third or fourth component, or by making the number of mixture components itself a free parameter, predicated upon some information theoretical criterion (Marin et al., 2005). Thus, in the example above, a third distribution might be appropriate if it was further suspected that there was group of abnormally high-achieving observers (e.g., individuals with extensive prior experience, or some physiological advantage). *N*-dimensional GMMs are not considered in the present work, however, as the size of a typical psychophysical dataset is generally insufficient to constrain so many free parameters.

### rSD

Same as *SD*, but applied recursively until no additional outliers are identified:

3 $$ \left\{\begin{array}{cc} |{x_{i}^{0}}| > \left( \bar{x}_{0} + \lambda\sigma_{0} \right)\\  |{x_{i}^{n}}| > \left( \bar{x}_{n} + \lambda\sigma_{n} \right). \end{array}\right. $$

This heuristic aims to solve the problem of masking (see above) by progressively peeling away the most extreme outliers. However, like *SD*, it remains intolerant to distributions that do not conform to the assumed Gaussian shape. In situations where samples are sparse or skewed, this approach is therefore liable to aggressively reject large quantities of genuine data (see Fig. 1b). Users typically attempt to compensate for this by using a relatively high criterion level, and/or by limiting the number of recursions (e.g., *λ* ≥ 3,*n*_max_ = 3).

### IQR

*x*_*i*_=outlier if it lies more than *λ* times the interquartile range from the median:

4 $$ |x_{i}| > \left( \tilde{x} + \lambda{}iqr \right). $$

This is a non-parametric analogue of the *SD* rule: substituting median and *iqr* for mean and standard deviation, respectively. Unlike *SD*, the key statistics are relatively robust: the breakdown points for $\tilde {x}$ and *iqr* are 50% and 25% (respectively), meaning that outliers can constitute up to 25% of the data before the estimated values start to become distorted (Rousseeuw & Croux, 1993). However, like *SD*, the *IQR* method only considers absolute deviation from the center. It therefore remains insensitive to any asymmetry in the sampling distribution (Fig. 1b, bottom).

### prctile

*x*_*i*_=outlier if it lies above the *λ*^th^ percentile, or below the (1 − *λ*)^th^:

5 $$ x_{i} > P_{\lambda} \qquad or \qquad x_{i} < P_{1-\lambda}. $$

This heuristic effectively ‘trims’ the data, rejecting the most extreme points, irrespective of their values. Unlike *IQR*, this approach is sensitive to asymmetry in the sampling distribution. However, it is otherwise crude in that it ignores any information contained in the spread of the data points. The *prctile* method also largely begs the question, since the experimenter must estimate, a priori, the number of outliers that will be observed. If *λ* is set incorrectly, genuine data may be excluded, or outliers missed.

### Tukey

*x*_*i*_=outlier if it lies more than *λ* times the iqr from the 25th/75th percentile:

6 $$ x_{i} > \left( P_{75} + \lambda{}iqr \right) \qquad or \qquad x_{i} < \left( P_{25} - \lambda{}iqr \right). $$

Popularized by the renowned statistician John W. Tukey, this heuristic, otherwise known as the ‘fence’ or ‘boxplot’ approach, attempts to combine the best features of the *IQR* and *prctile* methods. The information contained in the spread of data, *iqr*, is combined with the use of lower/upper quartile ‘fences’ that provide some sensitivity to asymmetry.

### MAD_*n*_

*x*_*i*_=outlier if it lies farther from the median than *λ* times the median absolute distance [MAD] of every point from the median:

7 $$ \left( \frac{ | x_{i} - \tilde{x} | } { MAD_{n} } \right) > \lambda \quad where \quad MAD_{n} = \underset{i=1:n}{\text{med}} | x_{i} - \underset{j=1:n}{\text{med}} x_{j} |, $$

Unlike the other non-parametric methods described previously, this heuristic uses MAD rather than *iqr* as the measure of spread. This makes it more robust, since the MAD statistic has the best possible breakdown point (50%, versus 25% for *iqr*). However, as with *IQR*, *MAD*_*n*_ assumes symmetry, only considering the absolute deviation of datapoints from the center.

### S_*n*_

*x*_*i*_=outlier if the median distance of *x*_*i*_ from all other points, is greater than *λ* times the median absolute distance of every point from every other point:

8 $$ \left( \frac{\text{med}_{j\neq i}|x_{i} - x_{j}| } { S_{n} } \right) \!>\! \lambda \quad where \quad S_{n} = c_{n} \underset{i=1:n}{\text{med}} \left\lbrace \underset{j\neq i}{\text{med}}|x_{i} \!- \!x_{j}| \right\rbrace , $$

where *c*_*n*_ is a bias correction factor for finite sample sizes (see Listing 1 for details). Introduced by Rousseeuw and Croux (1993), and *S*_*n*_ term, like MAD, is a maximally robust measure of spread. However, it differs from *MAD*_*n*_ in that *S*_*n*_ considers the typical distance between all data points, rather than measuring how far each point is from some central value. It therefore continues to provide a valid measure of spread even when the sampling distribution is asymmetric. The historic difficulty with *S*_*n*_ is its computational complexity. However, with modern computing power and the relatively small size of psychophysical datasets, processing times are negligible: on the order of milliseconds. For example, using an ordinary office PC, it takes just ∼4 ms to apply the Matlab code in Listing 1 to a vector of 100 data points.

## Comparison of techniques using simulated psychophysical observers

To assess the eight methods described in Section 2, each was applied to random samples of data, prelabeled either as ‘bad’ (should be excluded) or ‘good’ (should not be excluded). Rather than defining arbitrary sampling distributions for these two categories, and since their possible values are infinite, we instead simulated a specific situation, representative of a typical psychophysical scenario. Thus, a common situation is one in which the experimenter suspects some observers were not always/fully complying with the task instructions (e.g., due to boredom, fatigue, or malingering). The experimenter wishes to identify and exclude these individuals based on the statistically aberrant data they are likely to produce. This scenario was simulated as follows.

**Fig. 2:**
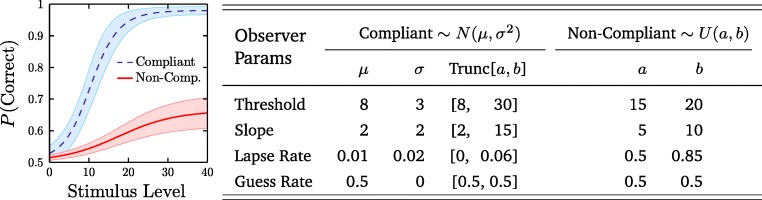
Mean [± 1 SD] psychometric functions for simulated observers. The shape of the function was logistic. The probability of responding correctly to a stimulus of magnitude *x* was therefore: $P(\text {Correct}) = G + (1 - L - G)(1 / [1+e^{-{\frac {x-T}{S}}}])$. The Guess Rate, *G*, was fixed at 50%. The other three parameters—threshold (*T*), slope (*S*), and lapse rate (*L*)—varied randomly between observers, according to either a truncated Gaussian distribution (compliant observers) or a uniform distribution (non-compliant observers). See table for exact values for the two distributions. For more information on psychometric functions and the four parameters employed here, see Klein (2001)

**Fig. 3:**
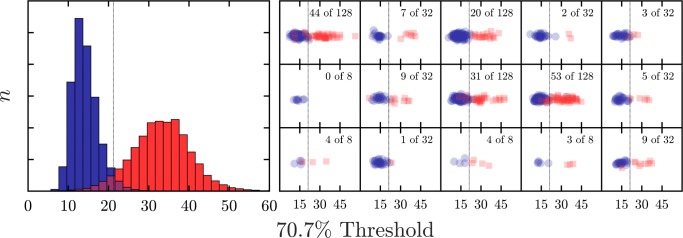
Data from simulated observers. (*left*) Sampling distributions for the 70.7% Threshold statistic, generated by simulation, using the psychometric functions in Fig. 2. (*right*) Example samples, with variable sample size 〈8,32,128〉 and proportion of non-compliant observers (0 to 50%). For every sample, each of the eight methods of outlier detection in Section 2 was applied, and its performance recorded. *Dashed vertical line* shows the ideal unbiased classifier, for which: hit rate = 0.97, false alarm rate = 0.05

Each simulated observer consisted of a randomly generated psychometric function (Fig. 2). Non-compliant observers had psychometric functions that tended to exhibit elevated thresholds, slopes, and lapse rates. They were thus more likely to produce statistically outlying data points (specifically: estimates of 70.7% threshold; Fig. 3, red bars). Compliant observers had psychometric functions lower with lower (better) thresholds, slopes, and lapse rates, and produced the main distribution of ‘good’ data (Fig. 3, blue bars). The Guess Rate of all function was fixed at 50%, reflected a typical two-alternative forced-choice [2AFC] paradigm (Macmillan & Creelman, 2005).

Using these psychometric functions, response data for individual trials were generated, and the resultant sequence of trial-by-trial responses were used to estimate perceptual thresholds (or: just noticeable differences), exactly as one would with a human participant. Specifically, on each trial a stochastic (correct/incorrect) response was generated, where the probability of responding correctly was determined by evaluating the psychometric function at the current stimulus magnitude (see Fig. 2). After each response, stimulus magnitude was varied according to a 2-down 1-up transformed staircase (Levitt, 1971). The experiment terminated after eight staircase reversals, and the final threshold was computed by mean-averaging the final four reversals. Figure 3 shows the resultant histogram of thresholds across a large number of simulated observers. For further details regarding the method simulation, the raw Matlab source code can be found at: https://github.com/petejonze/psychosim.

Simulations were repeated using varying sample sizes and varying proportions of non-compliant observers. Possible sample sizes, *n*, took the values 〈8,32,128〉, representing small, medium, and large psychophysical cohorts. The proportion of Non-Compliant observers varied from 0 to 50% of *n*, in integer steps (i.e., 〈0,1,...,16〉, when *n*= 32). This yielded a total of 54 unique conditions (sample size x proportion non-compliant), each of which was independently simulated 2000 times and the results mean-averaged to minimize error. Note that the use of 1999 repetitions is typical of such Monte Carlo simulations, and none of the present conclusions would be expected to change if this number were increased.

The outcome measures were: hit rate and false alarm rate (i.e., those signal detection theoretic performance metrics that characterize the sensitivity and specificity of a classifier (Macmillan & Creelman, 2005)), and robustness (i.e., how great a proportion of non-compliant observers could be tolerated, before performance deteriorated precipitously).

Note that non-compliance is not the only process that may give rise to statistical outliers in psychophysical datasets. Some processes, such as transcription errors of technical faults, can lead to missing values, or values so extreme that they may be relatively trivial to identify. Other processes can cause outliers to be distributed both above and below the main body of data (imagine, for example, if a test of color discrimination was applied to a broad population of people, including both dichromats and tetrachromats, who would be expected to score systematically worse/better than normal, respectively). In short, the present simulations were intended to be representative, not comprehensive, and a prudent reader may wish to modify the present code (see above for hyperlink) to simulate the exact dynamics of their particular experiment, or to assess novel methods of outlier detection. Also note that while the proportion of non-compliant observers was allowed to range from 0 to 50%, values greater than 5% would generally be considered extremely high in a population of healthy, well-motivated adults. Higher rates of non-compliance are not uncommon, however, when working with clinical or developmental datasets (Jones et al., 2015).

### Results and discussion

**Fig. 4:**
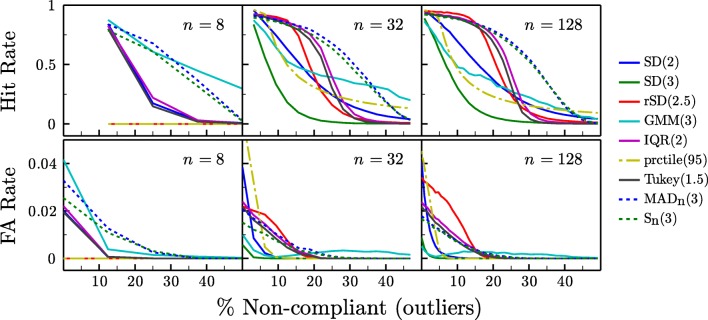
Simulation results. The eight classifiers described in Section 2 were used to distinguish between random samples of ‘compliant’ and ‘non-compliant’ simulated observers (see Fig. 3). *Numbers in parentheses* indicate the criterion level, *λ*, used by each classifier

The results of the simulations are shown in Fig. 4. We begin by considering only the case where *n*= 32 (Fig. 4, middle column)—a relatively typical sample size for behavioral experiments—before considering the effect of sample size.

In general, the *SD* rule proved poor. When *λ*= 3, it was excessively conservative—seldom exhibiting false alarms, but often failing to identify non-compliant observers [NCOs], particularly when the proportion of NCOs was large (low hit-rate/sensitivity). The use of a more liberal exclusion criterion (*λ*= 2) improved hit rates, but at the expense of a higher false alarm rate (low specificity), particularly when the number of outliers was low. Furthermore, even when *λ*= 2, *SD* still continued to exhibit a generally lower hit rate than most other methods.

The modified *GMM* rule (implemented here with one additional Gaussian component only, and constrained to have a mean greater than the 75^*th*^ percentile of a simple unimodal Gaussian fit) performed similarly to *SD*_*λ*= 3_, but exhibited greater robustness (i.e., a less rapid decline in hit rate as %NCOs increased). While the *rSD* rule generally exhibited high hit rates, but also high false alarm rates and a relatively steep decline in hit rates when %NCOs > 10*%*. Compared to the non-parametric methods, however, all of the *SD*-based rules generally performed poorly; only offering consistent advantages over the *prctile* rule: the performance of which was entirely dependent on the predefined exclusion rate matched the true number of outliers exactly. The only exception might be if the expected number of outliers was extremely low, in which case the *SD*_*λ*= 3_ rule might be considered sufficient, and may even be desirable if the cost associated with false alarms was exceptionally high.

The two *iqr*-based methods, *IQR* and *Tukey*, exhibited high hit rates when the number of outliers was low (≤ 20*%*). However, as expected, hit rates deteriorated markedly as the number of outliers increased (i.e., in accordance with the 25% breakdown point for *iqr*). False alarm rates were also somewhat higher overall than *S*_*n*_.

The two median-absolute-deviation based methods, *MAD*_*n*_ and *S*_*n*_, were as sensitive as all other methods when outliers were few (≤ 20*%*), and were more robust than the *iqr* methods—continuing to exhibit high hit rates and few false alarms even when faced with large numbers of outliers. Compared to each other, *MAD*_*n*_ and *S*_*n*_ performed similarly. However, the S_**n**_ statistic exhibited slightly fewer false alarms. It also makes no assumption of symmetry, and so ought to be superior in situations where the sampling distribution is heavily skewed.

We turn now to how sample size affected performance. With large samples (*n*= 128), the pattern was largely unchanged from the medium sample-size case (*n*= 32) except that *rSD* exhibited a marked increase in false alarms, making it an unappealing option. Again, *S*_*n*_ was generally superior, except in terms of a slight elevation in false alarms at very low %NCO (relative to the very conservative *SD*_*λ*= 3_ rule). With small samples (*n*= 8), the *prctile* and *rSD* methods became uniformly inoperable, while most other methods were generally unable to identify more than a single outlier. The *MAD*_*n*_ and *S*_*n*_ methods, however, remained relatively robust: exhibiting only a modest decrement in hit rates, though they did exhibit an elevated false alarm rate when there were few/no outliers. It may be that the latter could be rectified by increasing the criterion, *λ*, as a function of *n*, however this was not investigated. The *GMM* method also performed relatively well overall, but was only more sensitive than *MAD*_*n*_ or *S*_*n*_ when the proportion of outliers was extremely high (> 33*%*).

## Conclusions

Of the eight methods considered, *S*_*n*_ performed the best overall. It exhibited a high hit rate across all sample sizes, maintained a relative low false alarm rate, and was highly robust—able to cope even with very large numbers of outliers and/or very small sample sizes. Specific situations were observed in which other heuristics performed as-well-as, or even marginally better than, *S*_***n***_. For example, when sample sizes were large (*n*≥ 32) and the proportion of outliers few (< 25*%*) the non-parametric *IQR*/*Tukey* rules exhibit similar hit rates to *S*_*n*_, and only slightly more false alarms. Likewise, a conservative *SD* rule (*λ*= 3) proved sufficient to isolate extremely small numbers of outliers in large or midsized samples. In general though, alternative heuristics were generally no better than *S*_*n*_ in most circumstances, and failed precipitously in others (e.g., when the sample size was small or the proportion of outliers large). The *MAD*_*n*_ heuristic, which is closely related to *S*_*n*_, proved almost as strong, and can also be considered a good method for identifying outliers, as suggested previously by others (Leys et al., 2013). However, as discussed in Section 2 and elsewhere (Rousseeuw & Croux, 1993), the *MAD*_*n*_ statistic assumes a symmetric sampling distribution, and so would not be expected to perform as well in situations where the sampling distribution is very heavily skewed (e.g., when dealing with reaction time data (Ratcliff, 1993)). The popular *SD* metric and its derivatives proved poor in nearly all circumstances, and should never be used without independent justification (e.g., if real-time processing of extremely large datasets were required, at which point the computational overheads of *S*_*n*_ might become a non-trivial constraint).

In short, of the methods considered here, *S*_*n*_ appears to provide the best single means of identifying statistical outliers when the underlying sampling distribution is unknown. In the absence of countervailing reasons, it should therefore be considered the ‘default’ choice for researchers, and may be of particular benefit to those working with small or irregular populations such as children, animals, or clinical cohorts. Matlab code for computing *S*_***n***_ is provided in Listing 1. Many of the methods described here are also supported by various ‘robust statistics’ packages for R (Rousseeuw et al., 2009; Wilcox, 2012) and Matlab (Verboven & Hubert, 2005).

### On the ethics and practicalities of excluding statistical outliers

Excluding statistical outliers is often regarded as poor practice. Unless data could not possibly have arisen otherwise, we cannot generally be certain that any outliers were generated by some qualitatively distinct process (e.g., a subset of non-compliant or physiologically abnormal observers), and that they are not simply the tail end of a single, unitary population. By segregating such values, real and potentially interesting individual differences in ability may go unreported, and in the worst case the process of outlier exclusion can be manipulated to support weak or erroneous conclusions.

As shown in Section 2, however, the exclusion of statistical outliers can sometimes be preferable to reporting fundamentally misleading results. Automated methods of statistical outlier identification should never be used blindly though, and they are not a replacement for common sense. Where feasible, data points identified as statistical outliers should only be excluded in the presence of independent corroboration (e.g., experimenter observations), and the rates and criteria of exclusion should be articulated clearly. Furthermore, best practice dictates that when outliers are excluded, they should continue to be reported (e.g., graphically, and/or through independent analyses), and it should be confirmed whether any of the study’s conclusions are contingent on their exclusion. Thus, an example statement from a study’s Methods section might read as follows (NB: Supplemental Material not given):

Data from two participants (8.3%) were excluded post hoc on the grounds that they: (i) were observed to be inattentive and restless during testing; (ii) produced statistically outlying results ([med_*j*≠*i*_|*x*_*i*_ − *x*_*j*_|]/*S*_*n*_ > 3; see Rousseeuw & Croux, 1993); and (iii) exhibited high error rates (> 10*%*) on suprahthreshold (false-negative) catch trials. No other participants met any of these three criteria. Raw data from the two excluded participants are still displayed in relevant figures, but were not included in any analyses or descriptive statistics. The findings of the present study were unchanged if the reported analyses were repeated with these two participants included, with one minor exception (see Supplemental Material for details).

## References

[CR1] Cousineau D, Chartier S (2010). Outliers detection and treatment: a review. International Journal of Psychological Research.

[CR2] Huber PJ (2011). International Encyclopedia of Statistical Science, chap. Robust Statistics.

[CR3] Jones PR, Kalwarowsky S, Braddick OJ, Atkinson J, Nardini M (2015). Optimizing the rapid measurement of detection thresholds in infants. Journal of Vision.

[CR4] Klein SA (2001). Measuring estimating, and understanding the psychometric function. A commentary. Attention, Perception, & Psychophysics.

[CR5] Levitt H (1971). Transformed up-down methods in psychoacoustics. The Journal of the Acoustical Society of America.

[CR6] Leys C, Ley C, Klein O, Bernard P, Licata L (2013). Detecting outliers: Do not use standard deviation around the mean, use absolute deviation around the median. Journal of Experimental Social Psychology.

[CR7] Macmillan NA, Creelman CD (2005). Detection theory: A user’s guide.

[CR8] Marin, J.-M., Mengersen, K., & Robert, C.P. (2005). Bayesian modelling and inference on mixtures of distributions. In D. Dey, & C. Rao (Eds.) *Essential Bayesian Models* (pp. 459–507). Amsterdam: Elsevier.

[CR9] Osborne JW, Overbay A (2004). The power of outliers (and why researchers should always check for them). Practical assessment, Research & Evaluation.

[CR10] Ratcliff R (1993). Methods for dealing with reaction time outliers. Psychological Bulletin.

[CR11] Rousseeuw, P., et al. (2009). Robustbase: basic robust statistics. R package version 0.4-5. http://CRAN.R-project.org/package=robustbase.

[CR12] Rousseeuw PJ, Croux C (1993). Alternatives to the median absolute deviation. Journal of the American Statistical association.

[CR13] Verboven S, Hubert M (2005). Libra: a MATLAB library for robust analysis. Chemometrics and Intelligent Laboratory Systems.

[CR14] Wilcox RR (2012). Introduction to Robust Estimation and Hypothesis Testing.

